# User-friendly solutions for microarray quality control and pre-processing on ArrayAnalysis.org

**DOI:** 10.1093/nar/gkt293

**Published:** 2013-04-24

**Authors:** Lars M. T. Eijssen, Magali Jaillard, Michiel E. Adriaens, Stan Gaj, Philip J. de Groot, Michael Müller, Chris T. Evelo

**Affiliations:** ^1^Department of Bioinformatics—BiGCaT, Maastricht University, PO Box 616, 6200 MD Maastricht, The Netherlands, ^2^Department of Experimental Cardiology, Heart Failure Research Center, Academic Medical Center, University of Amsterdam, PO Box 22660, 1100 DD Amsterdam, The Netherlands, ^3^Department of Toxicogenomics, Maastricht University, PO Box 616, 6200 MD Maastricht, The Netherlands and ^4^Nutrition, Metabolism and Genomics Group, Division of Human Nutrition, Wageningen UR, PO Box 8129, 6700 AA Wageningen, The Netherlands

## Abstract

Quality control (QC) is crucial for any scientific method producing data. Applying adequate QC introduces new challenges in the genomics field where large amounts of data are produced with complex technologies. For DNA microarrays, specific algorithms for QC and pre-processing including normalization have been developed by the scientific community, especially for expression chips of the Affymetrix platform. Many of these have been implemented in the statistical scripting language R and are available from the Bioconductor repository. However, application is hampered by lack of integrative tools that can be used by users of any experience level. To fill this gap, we developed a freely available tool for QC and pre-processing of Affymetrix gene expression results, extending, integrating and harmonizing functionality of Bioconductor packages. The tool can be easily accessed through a wizard-like web portal at http://www.arrayanalysis.org or downloaded for local use in R. The portal provides extensive documentation, including user guides, interpretation help with real output illustrations and detailed technical documentation. It assists newcomers to the field in performing state-of-the-art QC and pre-processing while offering data analysts an integral open-source package. Providing the scientific community with this easily accessible tool will allow improving data quality and reuse and adoption of standards.

## INTRODUCTION

Development of standardized data processing methods has been important for the establishment of gene expression microarray technology. Generally accepted quality control (QC) and data processing methods have been made available, especially for expression chips of the Affymetrix platform (1). This gain of experience contrasts with the lack of an easy accessible QC and pre-processing tool, inducing a tendency among researchers to not use current knowledge, or even omitting or minimizing QC (2). Various companies, including Affymetrix, provide suites, such as the Affymetrix Expression Console Software. These are mostly proprietary software, not readily available to all researchers, and difficult to connect to or extend with additional functionality. Furthermore, they tend to lag behind the most recent developments in the field. Besides commercial tools, several open-source packages are available.

The Bioconductor repository of R libraries provides one of the most extensive, regularly updated and relied on public collections of microarray data QC and pre-processing methods (3,4). Their application, however, is not straightforward, as many different Bioconductor packages are available that all do part of the job, often depending on each other and often performing partially overlapping tasks. The output is not always easy to interpret and owing to the use of different graphical conventions by the different packages, it is hard to jointly understand results and obtain a good overview. Some Bioconductor packages generate limited reports of QC results when run by calls from within R. This is for example the case for the arrayQualityMetrics package, where the authors suggest the use of such a report in a web flow, and affyQCReport, which uses simpleAffy functionality (5,6). The affylmGUI and oneChannelGUI packages are accessible through a graphical user interface rendering several QC related images, but they still require local installation of R (7,8). In summary, these open-source solutions lack overall integration and are generally not easy to use for non-specialists.

QC of chip data is important and will remain so in future, even with next-generation sequencing methods becoming the state-of-the-art technology. Many laboratories have extensive experience with microarray technology, and facilities are widespread. For some applications, like pilot studies or large studies involving huge amounts of samples, relatively low cost and less data-intensive microarrays are likely to remain a method of choice. More importantly, the joint evaluation of new experimental results with already published data sets has become pivotal in modern integrative systems biology research (9). To support this, most journals require submission of data to online repositories such as ArrayExpress or Gene Expression Omnibus (GEO), which already contain vast amounts of microarray data (10,11). This means re-evaluation and re-analysis of that data will remain relevant (12). In this regard, we identified two caveats: (i) QC has not always been applied to its full extent on original publication of the data and (ii) differences in data analysis approaches and improvements in these approaches since publication require reprocessing data sets in a uniform way using the latest methods. The web portal and tool described here will allow for a swift application of standardized QC, normalization and re-annotation with respect to the latest genome builds.

## MATERIALS AND METHODS

We designed a web portal dedicated to integrated QC and pre-processing of Affymetrix expression chips, implementing a wizard-like web tool and offering online documentation. Our tool is the result of a joint effort combining, improving and extending functionalities of scripts from the BiGCaT department and QC and pre-processing scripts called by the MadMax server hosted by the Nutrition, Metabolism and Genomics laboratory of Wageningen University (13). The tool has been implemented in R with use of existing libraries from the Bioconductor repository as shown in Table 1 and data types defined by the affy library (3,4,14). The QC images have been adapted with a focus on producing more comprehensible and coherent results and in a more consistent format, adding new plots where needed. Furthermore, custom CDF re-annotations from the Brainarray website (15) and gene annotations from the Ensembl BioMart web resource (16,17) are incorporated.

**Table 1.** Overview of the four categories of QC results produced by ArrayAnalysis.org

Category	Graphs and tables computed on raw data	R/Bioconductor packages
Sample quality	Sample prep controls^a^	simpleaffy, yaqcaffy
	3′/5′ for b-actin and GAPDH^a^	simpleaffy
	RNA degradation plot	affy
Hybridization and overall signal quality	Spike-in hybrid. controls^a^	simpleaffy
	Background intensity^a^	simpleaffy
	Percentage present^a^	simpleaffy
	Present/Marg./Absent calls	simpleaffy
	Pos/Neg control distribution	affyQCReport
	All Affymetrix controls	affy, ArrayTools
Signal comparability and bias diagnostic	Scale factors^a^	simpleaffy
	Boxplot of log-intensity^b^	affy
	Density histogram^b^	affy
	MA plot^b^	affy
	Array reference layout	affy
	Pos/Neg controls COI plot	affyQCReport
	2D images	affy, affyPLM
	NUSE plot	affyPLM
	RLE plot	affyPLM
Array correlation	Correlation plot^b^	affy, gplots
	Hierarchical clustering dendrogram^b^	affy, bioDist
	PCA plot^b^	affy
Summary	Summary table	simpleaffy, yaqcaffy

Twenty plots and one table, classified into four main categories, are generated to assess the quality of the microarray data set. A summary table is composed to give an overview of the quality indicators marked by ‘a’. Six plots, marked by ‘b’, are recomputed after pre-processing the data to evaluate the correction of present artifacts by the normalization. Functionalities from the following Bioconductor libraries are adapted, extended and integrated within the tool: affy, affycomp, affypdnn, affyPLM, affyQCReport, ArrayTools, bioDist, biomaRt, gcrma, gdata, gplots, plier, RColorBrewer, simpleaffy, yaqcaffy. Note that the calculations using the gcrma, plier, simpleaffy and yaqcaffy packages support only the chip types supported by these packages; in case a requested image cannot be constructed, e.g. because of the chip type, the plot is omitted, and a warning is produced.

## RESULTS

We have built a user-friendly web portal that combines the powerful up-to-date functionalities of Bioconductor packages with the ease of use of a wizard-like interface and the automated generation of a customizable and integrated report. This serves less-experienced users to apply up-to-date QC and provides data analyst with an integrated tool and code base.

**Figure 1.**
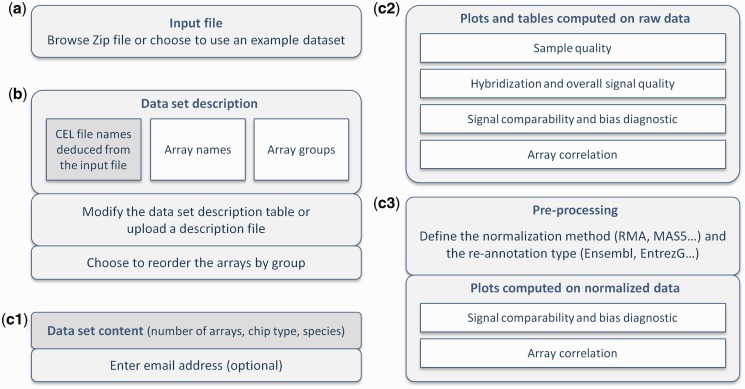
Schematic representation of the three input forms on the web portal composing the input wizard of ArrayAnalysis.org. (**a**) Upload of a ZIP file with CEL (or zipped CEL) files. (**b**) Definition of custom sample names and experimental grouping by either uploading a description file or completing the form, in which the CEL file names are prefilled based on the data set uploaded. (**c**) Selection of plots and computations to be returned: (c1) shows the detected array type, species and number of arrays in the data set and asks for an optional email address, (c2) selects elements in the four categories of plots and indicators applied to the raw data and (c3) defines the pre-processing steps and the plots evaluating these steps. Default settings will depend on the chip type of the uploaded CEL files and may be changed by the user.

On initiating a run on the ArrayAnalysis.org portal, the user is presented a three-stage wizard as described in Figure 1. The user is guided to upload the required data—a standard archive file (ZIP) containing the raw data (CEL files) and an optional description of the data set. After data upload, the user can indicate which computations and plots are to be returned, including setting preferences for the normalization and custom CDF re-annotation steps, for each of which a suitable default is provided. On completion, the portal presents an integrated report containing all the requested QC images, an archive with these images and tabular results, the normalized data and a log file that includes generated messages and an overview of the chosen settings. Results are displayed on screen, and links to result files are optionally sent by email. The web portal is free and open to all users, and there is no login requirement.

**Figure 2.**
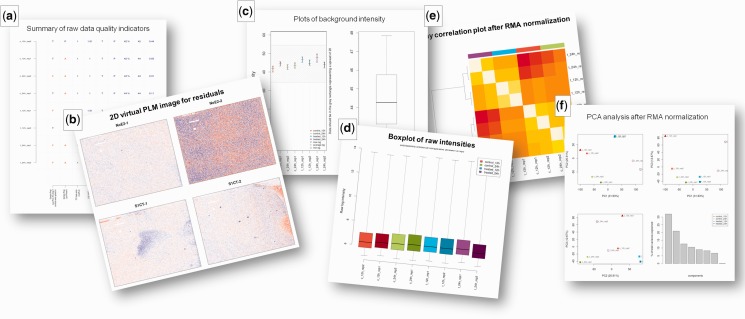
Sample of output images provided by the ArrayAnalysis.org QC tool. (**a**) Summary table of quality indicators; the indicator value is colored blue when within and red when out of the recommended cut-offs. (**b**) 2D image of the probe level model (PLM) residuals; this plot helps in the visualization of deviating regions on the chips. (**c**) Background intensity plot; a gray rectangle represents the maximal allowed spread. (**d**) Boxplot of raw data; this plot is also computed after normalization. (**e**) Array correlation plot after normalization; a color code of experimental groups eases the interpretation of the plot. (**f**) PCA analysis; 2D projections of the samples on the three principal components and histogram of explained variances by all components, ordered by decreasing percentage of total variance explained. The online documentation at ArrayAnalysis.org discusses all images produced and their interpretation in detail.

The first set of QC plots is computed on raw data and aims to give insight in the sample quality, the quality of the hybridizations and the overall signals. Furthermore, it evaluates comparability of signal strength and distribution within and between arrays, detecting deviating arrays (bias diagnostic), and assesses the correlation and grouping of samples based on the numeric array data. In those cases where criteria have been defined by Affymetrix or in the literature, the plots indicate whether these are met (18). To support interpretation, samples are consistently colored (and ordered if this option is selected) by experimental groups and labeled with user-provided custom names (see Figure 1b). Figure 2 shows several examples of output images. A complete description of the output is available in the online documentation.

The next set of QC plots is computed for pre-processed (annotated and normalized) data and allows evaluating the performance of the normalization (c.f. Table 1). Also, an annotated tab-delimited text file of normalized expression values is generated, where RMA, GC-RMA, MAS5 or PLIER can be selected as algorithms (19–21). Besides the standard Affymetrix annotation files, the tool facilitates the use of updated annotation files from the Brainarray laboratory (custom CDFs) to link data to targets (15). These annotation files re-annotate all probes based on a selected up-to-date database of choice and then regroup the probes into probe sets targeting unique genes.

Our tool is documented extensively in several ways. The web portal has mouse-over help tips for each item on the input forms, and it offers user guides and a guide for local installation. Additionally, a concise description is provided that helps interpretation of all plots and statistics produced. Each plot description ends with a link to the technical details of the custom-made R function invoked, where we document input and output parameters and defaults in a structured way, supporting developers. A bug tracker allows users to report problems or make feature requests and to check known issues. The portal also offers three example data sets, one for each of the two main generations of Affymetrix arrays (perfect match–mismatch arrays and perfect match only arrays) and one using custom arrays. These example sets are based on data published in the ArrayExpress repository (10). Set 1 uses the U133plus2.0 array with perfect match and mismatch probes (subset of E-GEOD-11352) (22); set 2 uses the HuGene-1.0-st array with perfect match probes only (E-GEOD-26747) (23); set 3 uses the custom-made NuGOMm1a520177 chip type, developed by the Nutrigenomics Organisation (http://www.nugo.org, subset of E-MTAB-601) (24,25). These data sets can be used by new users to try out the tool and to study the reports produced.

**Figure 3.**
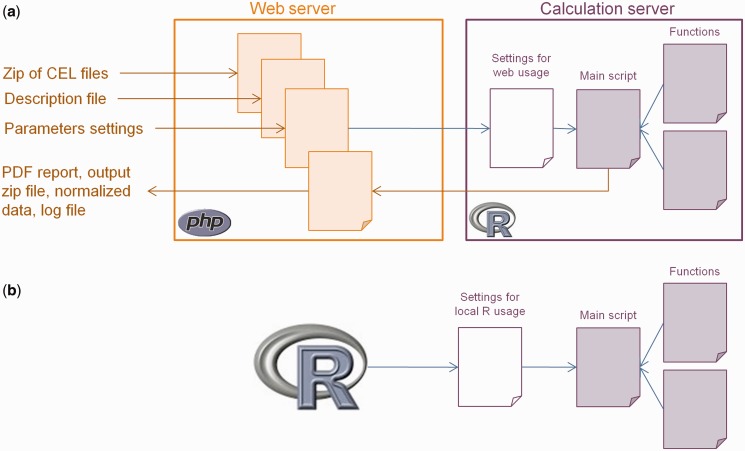
Structure of the ArrayAnalysis.org QC tool. (**a**) The ArrayAnalysis.org web server manages the input files and parameters and sends them to a distant calculation server using ssh2 and scp protocols. The R script runs on the calculation server and generates output images and tables. These output files are copied back to the web server, and a QC report and an archive are created from these files and displayed on the screen, together with a link to the normalized data and a log file of the run. (**b**) The package has a modular setup, which allows both calls via the web portal and local calls, while still using the same core functional code (represented as purple files), facilitating the implementation of updates. Once the settings and input files are correctly registered—this procedure differs for the web and local calls—the core script starts with loading the raw data and assigning sample names and experimental groups when provided. Then, the main script calls custom functions to compute QC plots and pre-process the data, which are stored in separate R scripts.

ArrayAnalysis.org gathers the input parameters in a single command sent to a remote calculation server, along with the input files, and collects the output files. This facilitates use of different servers or cloud usage. The core R functions are stored on the calculation server as shown in Figure 3a. Alternatively, the scripts can be installed locally and called directly from R on machines running R from version 2.12.0 upwards. For local use, we provide a main wrapper function for R (Figure 3b), which is available from a download page, which also provides a link to all source code on a subversion server. Functionality is distributed over scripts in a way that supports the implementation of updates.

## DISCUSSION

Availability of an easily and freely accessible tool that performs extensive state-of-the-art QC and that can be applied by users of all experience levels is important for the evaluation of both new data sets and already published ones to be used in integrated or comparative analyses. Approaches that offer an intuitive Affymetrix QC procedure on the web are scarce and not always updated or maintained. Some initiatives were launched to more generally ease the use of Bioconductor packages for QC of Affymetrix chips through web portals. RACE produces several QC images, but does not support recent chip types or updated annotations (26). AMarge offers a compact input form and returns a set of images (27). This project was published some years ago, but the website is not producing results anymore. Like RACE, it does not generate an integrated report. AffyGCQC implements some images of Affymetrix QC criteria and outlier detection for older chip types, for which it requires files already processed by the Affymetrix GCOS package as input (28). SmudgeMiner focuses on spatial biases only (29). To our best knowledge, there is currently no other QC tool besides ours that offers a web interface to R and Bioconductor functionalities to automatically generate a standardized QC report containing uniformly customized images. Our portal and tool are already in active use, being used by scientists all over the world. Furthermore, by its construction, ArrayAnalysis.org has been designed to be readily extended with further modules, e.g. handling other types of microarrays or performing further steps in the analytical process. Several of these modules are currently being built by us and our partners.

With the establishment of public data repositories, it has been understood that upload of additional information is needed to effectively use public genomics data. This has resulted in the strict requirement to provide a study description in MIAME compliant format along with the raw and processed data (30). We think that the availability of QC results is equally essential to properly judge the data set, and we propose to extend requirements to the upload of a well-defined minimal set of QC results when submitting data to a public repository. This ensures confirmation of data quality before publication of findings based on these data in a scientific paper. Also, it facilitates the selection of published data sets or arrays within those for integrative analyses or evaluation of new findings. Availability of a tool that brings together established methods and that every researcher can access and use, not only makes such requirements feasible but can also support the process of setting and adopting standards for upload of QC results, where ArrayAnalysis.org can fulfill a guiding role. Its functionality can also be directly incorporated by integrative systems biology tools that perform study capturing, data processing and storage, and prepare data for submission, such as dbNP (31).

In conclusion, ArrayAnalysis.org provides both wet-lab scientists and bioinformaticians with a powerful, easy-accessible and freely available tool for the QC and pre-processing of Affymetrix expression sets. Availability of our tool and web portal will assist researchers in applying and interpreting extensive chip QC, annotation and normalization, improving data quality and streamlining swift evaluation of multiple data sets for comparative analyses. Hereby, we aim to encourage researchers to apply state-of-the-art methods and to reuse already available data. We advocate that the complementary upload of a specified minimal set of QC endpoints should become mandatory when submitting data to public repositories. ArrayAnalysis.org can serve as a starting point for the design, implementation and dissemination of such a standardized QC approach.
